# Dietary L-glutamate modulates intestinal mucosal immunity of juvenile hybrid striped bass (*Morone saxatilis* ♀ × *Morone chrysops* ♂)

**DOI:** 10.3389/fimmu.2025.1575644

**Published:** 2025-04-10

**Authors:** Karina L. Hissen, Wenliang He, Guoyao Wu, Michael F. Criscitiello

**Affiliations:** ^1^ Comparative Immunogenetics Laboratory, Department of Veterinary Pathobiology, Texas A&M University, College Station, TX, United States; ^2^ Amino Acids Laboratory, Department of Animal Science, Texas A&M University, College Station, TX, United States; ^3^ Department of Microbial Pathogenesis and Immunology, College of Medicine, Texas A&M Health Science Center, Bryan, TX, United States

**Keywords:** intestinal mucosa, glutamate, nutrition, leukocytes, immune response, reactive oxygen species, hybrid striped bass, aquaculture

## Abstract

**Introduction:**

L-Glutamate is a conditionally essential amino acid, meaning it can become essential under specific conditions, like stress or disease. It is an abundant intracellular amino acid crucial in immune responses. Supplementation of feed with key amino acids, such as glutamate, can optimize growth and have other health benefits for production animals. Most research on dietary amino acid supplementation has focused on mammalian models, thus this research turned to hybrid striped bass, a teleost fish of growing importance to the aquaculture industry. The study investigated the effects of dietary supplementation with 0% or 5% glutamate in hybrid striped bass on intestinal mucosal immunity.

**Methods:**

The basal purified diet contained crystalline amino acids, including 3% L-glutamate. After an 8-week period of dietary supplementation with 5% glutamate followed by lipopolysaccharide stimulation, the intestinal mucosa was analyzed at the cellular and molecular levels to compare with the head kidney to assess potential changes in immune reactivity.

**Results:**

One week after lipopolysaccharide stimulation, glutamate supplementation enhanced (*P* < 0.05) the whole-body growth of fish without lipopolysaccharide challenge, total respiratory burst (the sum of O_2_^–^ and H_2_O_2_ production) in head kidney leukocytes, the net production of H_2_O_2_ in intestinal mucosal leukocytes, and upregulation of expression of mRNAs for IL-1β, TNF-α, and IgT in the gut mucosa.

**Discussion:**

Dietary supplementation with 5% L-glutamate may modulate intestinal mucosal immunity and improve growth in HSB to enhance disease resistance. Further research is needed to clarify the mechanism and cost-effective application.

## 1 Introduction

Dietary amino acids (AAs) are the building blocks of proteins. They are obtained through consuming myriad protein sources, and the AAs play a crucial role in the nutrition and health of animals (1, 2). Among these AAs, L-glutamate (Glu) is produced endogenously in tissues of animals, including mammals and fish such as hybrid striped bass (HSB) (3–5). Glu is synthesized *de novo* from (a) other AAs and α-ketoacids via transaminases, (b) the hydrolysis of L-glutamine by phosphate-activated glutaminase, and (c) ammonia plus α-ketoglutarate by Glu dehydrogenase (3, 6). Studies in young pigs have shown that a substantial portion of dietary Glu—up to 97% —is metabolized in the small intestine, predominantly by enterocytes (7). Notably, fish exhibit a remarkable capacity to utilize dietary protein as an energy source (8–10), suggesting a potential to strategically exploit Glu in their intestinal physiology and immunity. However, the metabolism of Glu in specific cells and tissues, as well as its dietary use in teleost fish, remains poorly understood.

Based on findings in neonatal pig enterocytes and activated rat macrophages, glucose is oxidized through the pentose cycle (15% and up to 30% respectively), a pathway efficient in producing NADPH (11, 12). NADPH is required for respiratory burst, a mechanism seen in phagocytic leukocytes that produce reactive oxygen species (ROS) to kill pathogens (13–15). In the intestinal mucosal leukocytes of HSB, glucose oxidation through the pentose cycle was at higher rates than glycolysis, approximately 42.4 and 10.7 nmol/2 h/10^6^ cells, respectively (16). It is here where Glu may play a role in modulating oxidant-antioxidant balance, because NADPH is used for the generation of superoxide anion (O_2_^–^) and hydrogen peroxide (H_2_O_2_) from O_2_ and of reduced glutathione (a potent antioxidant) from oxidized glutathione (12). Traditionally classified as a non-essential amino acid (NEAA) in animal nutrition, Glu has now been recognized as a functional amino acid due, in part, to its significant role in the immune function of terrestrial animals (3, 17–20). Initial attempts to investigate dietary glutamate were interrupted due to power failure. A fortuitous result from the accident was the observations that juvenile HSB supplemented with 1–5% glutamate had a 52–54% survival rate under decreased dissolved oxygen overnight and that 100% of these fish survived after abrupt water quality changes (21). In contrast, all HSB fed a glutamate-free diet died under conditions of the decreased dissolved oxygen or within 10 min after abrupt water quality changes (21). These results indicate that HSB’s endogenous glutamate synthesis was insufficient, and that dietary provision of adequate glutamate was necessary for its health and survival under stressful conditions.

Despite advances in the nutrition field, there is still a considerable gap in our understanding of how Glu affects the intestinal mucosa’s immunocytes (15). For fish, mucosal surfaces play a crucial role as the first line of defense against environmental pathogens, given the direct exposure to their aquatic environment (22). Gaining a deeper understanding of the fundamental principles of their immune systems could offer valuable insights into enhancing nutritional immune support in aquaculture (22–24).

Among animal protein sources, fish provide high-quality protein and other essential nutrients for human health (15, 25). As the United States shifts toward more sustainable fisheries and aquaculture practices, there is an increasing focus on fish immunity, especially in high-density, closed-system aquaculture such as those used for HSB. Over the past decade, HSB has been one of the fastest-growing animal production sectors in the United States (25–27). Therefore, understanding the optimal AA requirements in HSB is crucial for formulating ideal diets to enhance growth and disease resistance. High-density aquaculture has subjected fish to increased pathogen pressures, and infectious diseases can result in substantial economic losses estimated at up to $10 billion annually in global aquaculture (25). Thus, implementing effective fish health management strategies is imperative. This study proposes nutritional interventions to bolster immunity as a non-invasive and less labor-intensive alternative to traditional methods such as vaccination. The goal is to improve production efficiency by advancing our understanding of AA nutrition through immunomodulation in HSB.

Glu is one of the most abundant AAs in commonly used protein sources for aquafeeds—including fishmeal, poultry by-product meal, and soybean meal (28, 29)—highlighting the importance of understanding its role in teleost fish’s nutrition and immune function. The primary objective in this study is to explore the influence of dietary Glu on the intestinal mucosal leukocytes’ immune response in HSB. The hypothesis is that Glu supports the intestinal mucosal immunity of HSB by regulating respiratory burst (the production of O_2_^–^ and H_2_O_2_) in leukocytes to enhance immune responses in ways that can be exploited by feed optimization. These ROS kill pathogens in the innate immune response (14). The current study aims to employ purified diets containing crystalline AAs.

## 2 Materials and methods

This study (AUP # IACUC 2020-0322) was conducted following the approval by the Institutional Animal Care and Use Committee of Texas A&M University (College Station, TX, USA) on February 23, 2021 according to the Animal Welfare Act and Regulations of the United States Department of Agriculture.

### 2.1 HSB husbandry and sample collection

#### 2.1.1 Animals and housing

**Figure 1:**
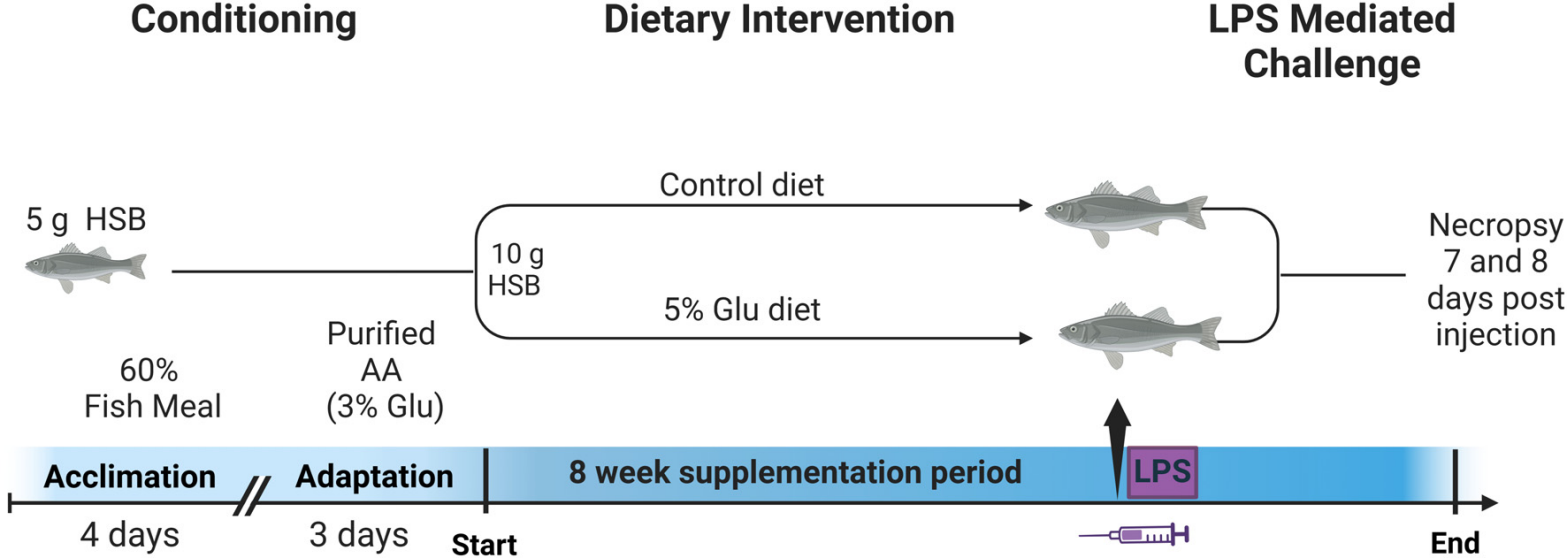
Experimental timeline for the animal feeding trial. The trial consisted of three phases: a conditioning phase, a dietary intervention period, and a 7-day post-LPS mediated challenge. The conditioning phase consisted of acclimating 5 g HSB from Keo Farms to the system with a 60% fishmeal diet (4 days) and the control diet (3 days) made in-house and fed to satiation twice daily. HSBs were fed until they reached a weight of 10 grams to start the 8-week supplementation period, where HSB were subjected to the control diet or the experimental diet containing an additional 5% Glu. After the 8-week supplementation period, HSB from each diet group were randomly selected to receive the intraperitoneal administration of 0.1 mL of RPMI medium containing either 0 (sham) or 100 µg LPS and were continually fed with their correspond diets. On Day 7 of post-LPS injection, all fish were weighed. On Days 7 and 8, fish (half of them on each day) were euthanized for tissue collection. Created in BioRender. Hissen, K. (2025) https://BioRender.com/p33v886.

Juvenile HSBs weighing approximately 5 grams were sourced from Keo Fish Farm (Keo, Arkansas, USA) and housed in a recirculating aquaculture system. The experimental setup included 12 tanks, each with four fish, containing 38 gallons of water maintained at 25–27°C. Aeration was provided by electromagnetic commercial air pumps (VEVOR, Amazon.com, Seattle, MA, USA) connected to air stones (2 per tank). The system utilized deionized water from a central reservoir, with water quality closely monitored through regular changes (30–50% daily) and salinity adjusted to 2–4 ppt using sea salt (Instant Ocean, Blacksburg, VA). Key water parameters, including salinity (2–3 ppt), pH (6.5–7.5), NH_4_^+^ (< 0.5 mg/L), nitrite (< 1 mg/L), nitrate (< 20 mg/L), and dissolved oxygen (6–8 ppm), were measured daily (except for dissolved oxygen) to ensure optimal and consistent conditions. Since water parameters were stable throughout the trial, dissolved oxygen was measured weekly. The tanks were maintained on a 12-hour light cycle from 8:00 AM to 8:00 PM. The fish were acclimated for one week in our system, where for the first four days, HSB were fed a 60% fishmeal diet made in-house and fed to satiation twice daily (3). For the following three days, the diet was switched to a purified formulation containing 3% Glu (**Table 1**), matched to the nutritional profile of 60% fishmeal (3). HSBs were fed until they reached a body weight of 10 grams to start the 8-week supplementation period. The experimental timeline is shown in **Figure 1**.

**Table 1:** The formulation and composition of the purified diets as fed during the 8-week supplementation period^a^.

Ingredients (g/kg)		Diets	
	Control	5% Glu
Fish oil^b^	110	110
Soy oil^c^	10	10
Dextrinized starch^d^	200	200
Cellulose^e^	139.925	120.205
Carboxymethyl cellulose (CMC)^f^	30	30
Vitamin premix^g^	1.055	1.055
Macromineral premix^h^	76.14	76.14
Micromineral pemix^i^	0.900	0.900
Amino acid (AA) mix (see below)^j^	360.78	380.5
Non-AA nitrogenous substances^k^	11.2	11.2
Water	60	60
Total	1000	1000
AA mix^j^	g/kg	g/kg
Arg	20.2	20.2
Asn	12.5	12.5
Asp	18.3	18.3
Cys	3.7	3.7
Gln	20.1	20.1
Gly	23.5	23.5
His	7.8	7.8
Ile	13.3	13.3
Leu	24.8	24.8
Lys-HCl	30.7	30.7
Met	10.9	10.9
Phe	12.8	12.8
Pro	20.6	20.6
Ser	14	14
Thr	13.8	13.8
Trp	3.9	3.9
Tyr	10.4	10.4
Val	16.7	16.7
Taurine	2.5	2.5
Ala	50.3	20
Glu	30	80

a Values are expressed on an as-fed basis.

b Fish oil (Paragon, Illinois, USA).

c Nutrioli pure soybean oil (Ragasa, N.L., Mexico).

d Maltodextrin (Amazon.com, Seattle, MA, USA).

e Microcrystaline cellulose 102 (Blue Diamond Growers, California, USA).

f Sodium carboxy methyl cellulose (Pro Supply Outlet, California, USA).

g Providing the following vitamins (mg/kg diet): retinyl (vitamin A) acetate, 23.06; cholecalciferol (vitamin D_3_), 20.24; DL-α-tocopheryl (vitamin E) acetate, 200; menadione (vitamin K_3_), 12; vitamin C, 300; DL-calcium pantothenate (vitamin B_5_), 109; myo-inositol, 150; niacin, 140; pyridoxine (vitamin B_6_), 30.4; vitamin B_2_; thiamine (vitamin B_1_) mononitrate, 32.6; biotin, 1.5; folic acid, 6; vitamin B_12_, 0.2.

h Providing the following macrominerals (g/kg diet): CaHPO_4_.2H_2_O, 33.7; NaCl, 15.3; MgSO_4_.7H_2_O. 14; and KCl, 13.14;

i Providing the following minerals (mg/kg diet): chromium(III) chloride, 7.3; CuSO_4_·5H_2_O, 35; FeSO_4_·7H_2_O, 498; MnSO_4_·4H_2_O, 82; Na_2_SeO_3_, 3; ZnSO_4_·7H_2_O, 258; sodium molybdate, 0.26; sodium fluoride 1.3; CoCl·6H_2_O, 5.2; KI, 7.8; and nickel chloride, 2.2.

j AA mixture (crystalline AAs; Ajinomoto, Tokyo, Japan).

k Providing the following substances (g/kg diet): choline chloride, 2.4; betaine, 5; inosine 5’-monophosphate, 3; creatine, 0.72; and carnitine, 0.08.

#### 2.1.2 Diets

Purified diets were formulated and made in the laboratory to evaluate the effect of dietary Glu on intestinal mucosal immunity in HSB and ensure precise nutrient manipulation by eliminating confounding variables (**Table 1**). All AAs (except for glycine and taurine) used were in their L-form. The formulation of the experimental diets complied with the recommended requirements for energy, nutritionally essential AAs, fatty acids, vitamins, and minerals as specified by the National Research Council (NRC) for HSB (30). L-alanine was used as the isonitrogenous control in the control diet. Before use, all dry ingredients were thoroughly mixed, followed by adding fish oil and water to create a semi-moist dough. The diet dough was further mixed thoroughly for extrusion to form pellets using a screw extruder (Big Bite Meat Grinder, West Chester, OH) fitted with a 1/8-inch plate. The extruded pellets were dried at 37°C until both diets reached a dry matter content of 92.6% to ensure consistent nutrient delivery. The prepared pellets were stored at –20°C in plastic zip-lock bags until use.

#### 2.1.3 Experimental trial

Fish of about 10 grams were randomly assigned to one of 12 tanks per diet group. Feed pellets were manually distributed to each tank to ensure that HSB received their designated food during feeding sessions. In cases of fish mortality, the amount of feed provided to each tank was adjusted according to the number of surviving fish to maintain consistent feeding levels per fish. After the 8-week supplementation period, HSB from each diet group were randomly selected to receive the intraperitoneal administration of 0.1 mL of RPMI medium containing either 0 (sham) or 100 µg of 2,4,6-trinitrophenyl hapten conjugated lipopolysaccharide (LPS; Santa Cruz Biotechnology, Santa Cruz, CA). On Day 7 post-sham or LPS injection, all fish were weighed. On Days 7 and 8, fish (half of them on each day) were euthanized for tissue collection, as shown in **Figure 1**.

#### 2.1.4 Sample collection

On the day of necropsies, tissue sampling was performed 4 h post-feeding. Specifically, whole blood (~0.5 mL) was obtained from the caudal vein of HSB using a 1-mL heparinized syringe and immediately centrifuged at 10,000 × *g* for 1 min to obtain the plasma. The latter was rapidly placed in liquid nitrogen and stored at –80°C until biochemical analyses. After blood sampling, HSBs were euthanized using freshly prepared 140 ppm MS-222 buffered to pH 7.5 with an appropriate amount of sodium bicarbonate. Fish were considered unresponsive when they did not respond to touch for 1 min, and their gills did not move. Thereafter, the intestine was collected and then butterflied open to be washed with oxygenated (95% O_2_/5% CO_2_) Ca^+^-free Krebs–Henseleit bicarbonate buffer (KHB, pH 7.4; 119 mM NaCl, 4.8 mM KCl, 1.2 mM MgSO_4_, 1.2 mM KH_2_PO_4_, and 25 mM NaHCO_3_, pH 7.4) containing 20 mM Hepes (pH 7.4), 5 mM glucose and 0.5% of fatty acid-free bovine serum albumin (BSA). For the remainder of the article, this buffer was referred to as KHB-BSA. The intestinal mucosal tissue was obtained using a glass slide to gently scrape the mucosa from the gut submucosa. The head kidneys were collected by dissecting. A portion of each tissue was placed in the KHB-BSA buffer for the isolation of leukocytes, and another portion (the midgut in the case of the intestine) was preserved in RNAlater^®^ solution. After overnight standing at 4°C, the RNAlater^®^-preserved tissues were stored at –20°C until they were used for RNA extraction.

### 2.2 Biochemical assays

#### 2.2.1 ROS release from leukocytes

On Days 7 and 8 post-sham or LPS injection, intestinal mucosa samples were collected from 6 fish of the same tank and pooled as one sample in a 5 mL tube containing KHB-BSA buffer. This ensured that there were enough cells for biochemical measurements. Head-kidney samples from 6 fish were also pooled as one sample in a 5 mL tube with KHB-BSA buffer. Both types of tissues were then gently passed through a 3-μm mesh filter in KHB-BSA buffer.

Leukocytes were isolated using Ficoll-Hypaque (specific gravity = 1.077) and subsequently assessed for respiratory burst activity by measuring the release of O_2_^-^ and H_2_O_2_ in the presence or absence of the mitogens, phorbol myristate acetate (PMA) plus ionomycin (IONO), as outlined by Wu and Marliss (11). Briefly, the net release of O_2_^-^ by leukocytes was quantified using the SOD-inhibitable reduction of ferricytochrome c (11, 31). Cells were incubated for 30 min at 26°C in 1 mL of two different reaction mixtures, each containing 0.5% BSA, 5 mM glucose, 80 µM ferricytochrome c, and either 0 or 40 µg SOD, with or without phorbol myristate acetate (PMA, the final concentration of 500 ng/mL) plus ionomycin (IONO, the final concentration of 7.5 ng/mL). PMA and IONO are mitogens that activate protein kinase C. The absorbance of the supernatant at 550 nm was measured using a spectrophotometric plate reader (BioTek Synergy H1, Agilent Technologies, Santa Clara, CA). The amount of net O_2_^-^ released was calculated based on the number of cells per well, using the formula O_2_^-^ release (nmol/cells) = (A_sample_ – A_reference_) × 71.4 × 1.333/number of cells in an assay tube. The length of the light path in each 350-microliter well of the microplates used is assumed to be 1 cm.

For measuring the net release of H_2_O_2_, leukocytes were incubated at 26°C in 1 mL of KHB buffer (pH 7.4) containing 0.5% BSA, 5 mM glucose, 0.56 mM phenol red, and peroxidase (0.1 mg/mL), with or without PMA (500 ng/mL) plus IONO (7.5 ng/mL). After 30 min, the reaction was stopped by mixing the medium with 13.3 mM NaOH (11, 31). Standard curves were generated under the same conditions in quadruplicates.

#### 2.2.2 Analysis of AAs

Free AAs in the plasma were analyzed using a Waters Alliance HPLC system (Milford, MA, USA), employing precolumn derivatization with OPA as described by Dai et al. (32). Briefly, 20 µL of plasma samples were acidified with 20 µL of 1.5 mol/L HClO_4_ and vortexed. The acidified samples were mixed with 450 µL of HPLC-grade water, neutralized with 10 µL of 2 mol/L K_2_CO_3_, and vortexed again. Following neutralization, the samples were centrifuged at 10,000 × *g* for 1 min using an Eppendorf 5920R centrifuge, and the supernatant fluid was collected for AA analysis.

For the HPLC analysis, the AA standards (50 µM for each AA) and neutralized plasma samples were prepared in 2-mL glass vials containing 0.1 mL of 1.2% benzoic acid (an antimicrobial agent, prepared in saturated potassium borate), 0.1 mL of a 50 µM AA standard solution, and 1.4 mL of HPLC-grade water. The mixture was vortexed for 10 sec. An aliquot of 15 µL from this solution was mixed with 15 µL of the 30 mM OPA reagent for 1 min in the autosampler. The OPA-AA derivatives were separated on a Supelco 3-µm reverse-phase C_18_ column (150 mm × 4.6 mm ID, Sigma-Aldrich, St. Louis, Missouri) guarded by a Supelco 5-µm reverse-phase C_18_ column (50 mm × 4.6 mm ID) with a solvent gradient composed of Solution A (0.1 mM sodium acetate (pH 7.2), 9% methanol, and 0.5% tetrahydrofuran) and Solution B (methanol). Amino acid concentrations in samples were quantified relative to authentic standards using the Empower-3 Software (Waters, Milford, MA).

#### 2.2.3 Relative RNA expression

RNAlater^®^ RNA-preserved immune tissues, including head kidney and mid-gut mucosa, were homogenized separately for RNA extraction using the RNeasy^®^ 100 Mini Kit (Qiagen, Hilden, Germany) and TissueLyser II (Qiagen, Hilden, Germany) with 5-mm stainless steel beads. RNA concentration was measured using a NanoDrop^®^ ND-1000 Spectrophotometer (Thermo Fisher Scientific, Waltham, MA). Samples with a 260/280 ratio outside the range of 2.0 to 2.2 were excluded from downstream applications. Repeated samples were selected based on these quality parameters and higher RNA concentration. According to the manufacturer’s instructions, the first-strand cDNA synthesis was performed using the SuperScript™ III First-Strand Synthesis System (Thermo Fisher Scientific, Waltham, MA). 500 ng of RNA per sample was mixed in a 1:1 ratio with 50 ng/μL random hexamers and 50 µM oligo dT primers, then incubated at 65°C for 5 min to denature secondary structures.

The degenerate primers for IL-1β and TNF-α, used in this study were adopted from Jeon and Fast (33) and were validated through conventional PCR and gel electrophoresis. IgT (FM010886) (34) and EF-1α (AJ866727) (33) were also confirmed in various HSB tissues, ensuring the expected amplicon size (**Table 2**). Additionally, real-time quantitative PCR (RT-qPCR) was conducted using SYBR^®^ Green PCR Master Mix (Applied Biosystems, Waltham, MA) to determine primer efficiency before measuring the relative gene expression in all samples. Each 10-μL reaction contained 5 μL of SYBR master mix, 1 μL of 5 μM forward and reverse primers, and 4 μL of cDNA at a 1:100 dilution. The amplification protocol included 50°C for 2 min, followed by 95°C for 2 min for polymerase activation, then 40 cycles of 95°C for 15 sec (annealing), 60°C for 1 min (denaturing), and 72°C for 1 min (extending). A melting curve analysis was performed with a 0.5°C increment per cycle over 5 sec between 55 and 95°C. Relative gene expression was calculated using the Fu et al. method (35), with results normalized against the control-sham group using EF-1α (AJ866727) (33) as the reference gene. Briefly, normalization was performed by dividing each group’s mean value by the mean value for the control-sham group. Normalized values were expressed as fold changes relative to the control-sham group that was set to 1. The standard error of the mean (SEM) for normalized data was calculated by dividing the SEM of each group by the mean for the control-sham group. The normalized data are reported as mean ± SEM.

**Table 2:** Primer sequences of genes of interest and a reference gene for real-time qPCR.

Gene	Primer Name	Primer Sequence	Length (bp)	Ref.
Elongation factor 1α^a^	EF1α-F	CTTGACGGACACGTTCTTGA	151	(33)
	EF1α-R	GTGGAGACCGGTGTCCTGAA		
Interleukin β	IL1β-F	CAGACTGGCTTTGTCCACTG	77	
	IL1β-R	AGTCCTGCTGATTTGATCTACC		
Tumor necrosis factor α	TNFα-F	AACGATGGTGAAGAGGAAAG	80	
	TNFα-R	CCTATGGAGTCTGAGTAGCG		
Immunoglobulin T	IgT-F	TCACTTGGCAAATTGATGGA	143	(34)
	IgT-R	AGAACAGCGCACTTTGTTGA		

a Reference gene.

Primers were taken from the literature (see reference).

### 2.3 Histology

To assess goblet cell distribution, 3-mm × 3-mm proximal sections were collected from two fish per treatment group and fixed in Carnoy’s Solution (60% ethanol, 30% chloroform, and 10% glacial acetic acid) for 45 min (36–38). They were then rinsed with 70% ethanol to stop the fixation process and stored until sent to the Veterinary Medicine and Biomedical Science (VMBS) Histology Laboratory at Texas A&M. There, the tissues were embedded in paraffin and cut in 4-µm consecutive cross sections with a rotary microtome and placed on charged slides. Sections were stained with hematoxylin and eosin (H&E) and Alcian Blue, pH 2.5. With assistance from the digital pathology group, slides were scanned and analyzed using Concentriq by Proscia.

### 2.4 Statistical analysis

All data are presented as means ± SEM. Statistical analyses were conducted using two-way ANOVA as described by Assaad et al. (39), and other tests, including the paired t-test and three-way ANOVA, utilizing JMP^®^, Version 16.0.0 (SAS Institute Inc., Cary, NC). Pairwise comparisons were conducted using the Student-Newman-Keuls (SNK) test to identify significant differences among group means (40).

## 3 Results

### 3.1 Growth performance

LPS challenge decreased growth in HSB and this effect of LPS was attenuated by dietary Glu supplementation (**Table 3**). Specifically, in the absence of LPS treatment, the body weight and weight gain of HSB in the 5%-Glu group were 17% (P = 0.006) and 52% (P = 0.026) greater, respectively, than the 0% Glu group. Challenge with LPS reduced the body weight and weight gain of HSB by13% (P = 0.010) and 46% (P = 0.026), respectively, compared with the sham counterparts. Neither the body weight nor the weight gain of HSB differed (P > 0.05) between the 0% Glu + LPS and the 5% Glu + LPS groups, although the values for the the 5% Glu + LPS group were numerically 8% and 17% greater, respectively, than those for the 0% Glu + LPS group.

**Table 3:** Body weight and weight gain of HSB before and after the immune challenge.

	Control		5% Glutamate								
	Body Weight (g/fish)				^1^ *P*-value						
	Sham	LPS	Sham	LPS	Diet	LPS	Day	Diet × LPS	Diet × Day	LPS × Day	Diet × LPS × Day
Day 0	27.5 ± 1.0	26.7 ± 1.3	29.9 ± 1.6	28.5 ± 1.3	*0.006*	*0.010*	*<0.0001*	*0.370*	*0.383*	*0.108*	*0.561*
Day 7	33.4 ± 1.3^b^	30.4 ± 1.4^b^	38.9 ± 2.4^a^	32.8 ± 1.1^b^							
	Weight Gain (g/fish)				Diet		LPS			Diet × LPS	
	5.90 ± 0.46^b^	3.70 ± 0.56^b^	8.95 ± 1.04^a^	4.32 ± 0.84^b^	*0.026*		*<0.001*			*0.126*	

Values are means ± SEM, n = 6 tanks per treatment group.

^a-b^Means in a row without a common superscript letter differ (*P* < 0.05) as analyzed by the SNK multiple comparison test.^1^Probability values are indicated in italics.

### 3.2 AA concentration in plasma

The concentrations of only a few AAs in the plasma of HSB were affected by Glu supplementation or LPS challenge (**Table 4**). In the absence of LPS challenge, the concentrations of Glu, aspartate, asparagine, citrulline, arginine, and tyrosine in plasma were increased (P < 0.05) but those of taurine were decreased (P < 0.05) in HSB fed the 5% Glu diet. The concentrations of other AAs in plasma were not affected (P > 0.05) by diet. In response to LPS challenge, the concentrations of Glu, aspartate, asparagine, serine, and tyrosine in plasma were decreased (P < 0.05) but all measured AAs in plasma did not differ (P > 0.05) between the 0% Glu and the 5% Glu groups. There were interaction effects between Glu supplementation and LPS challenge in asparagine, citrulline, taurine, and tyrosine. Specifically, LPS challenge decreased (P < 0.05) the concentrations of asparagine, tyrosine, and citrulline in plasma in fish fed the 5% Glu diets but had no effect in fish fed the 0% Glu diets. In contrast, LPS challenge decreased (P < 0.05) the concentrations of taurine in plasma in fish fed the 0% Glu diets but had no effect in fish fed the 5% Glu diets.

**Table 4:** Concentrations (nmol/ml) of amino acids (AAs) in the plasma of HSB.

AA	Control		5%-Glu		^1^ *P*-value		
	SHAM	LPS	SHAM	LPS	Diet	LPS	Diet × LPS
Glu	120 ± 11^b^	86.0 ± 10.3^b^	228 ± 22^a^	114 ± 27^b^	*0.002*	*0.001*	*0.051*
Asp	103 ± 19^b^	67.6 ± 7.8^b^	157 ± 13^a^	71.4 ± 8.5^b^	*0.044*	*<0.001*	*0.076*
Asn	92.5 ± 9.8^b^	87.1 ± 10.0^b^	156 ± 26^a^	65.8 ± 4.5^b^	*0.174*	*0.006*	*0.012*
Ser	316 ± 36	278 ± 38	342 ± 37	223 ± 8.6	*0.668*	*0.026*	*0.223*
Gln	231 ± 15	237 ± 33	298 ± 47	231 ± 20	*0.344*	*0.342*	*0.257*
His	97.2 ± 11.4	172 ± 19	131 ± 27	138 ± 24	*0.996*	*0.074*	*0.135*
Gly	374 ± 40	314 ± 17	380 ± 36	343 ± 28	*0.578*	*0.146*	*0.713*
Thr	168 ± 17	156 ± 28	212 ± 44	151 ± 20	*0.505*	*0.232*	*0.410*
Cit	30.4 ± 13.0^b^	43.2 ± 8.6^b^	76.1 ± 15.5^a^	31.7 ± 6.7^b^	*0.155*	*0.189*	*0.024*
Arg	124 ± 10^b^	170 ± 10^a,b^	208 ± 26^a^	212 ± 30^a^	*0.009*	*0.252*	*0.327*
Tau	2080 ± 262^a^	772 ± 123^b^	1110 ± 228^b^	1380 ± 157^b^	*0.374*	*0.020*	*0.001*
Ala	881 ± 109	726 ± 46	786 ± 101	733 ± 103	*0.648*	*0.281*	*0.594*
Tyr	58.2 ± 8.7^b^	50.8 ± 5.6^b^	112 ± 10^a^	63.5 ± 7.4^b^	*0.001*	*0.003*	*0.023*
Trp	27.1 ± 2.8	29.9 ± 3.2	28.5 ± 5.0	28.8 ± 4.5	*0.962*	*0.698*	*0.759*
Met	104 ± 25	77.6 ± 11.1	104 ± 32	113 ± 15	*0.443*	*0.702*	*0.435*
Val	314 ± 53	232 ± 18	427 ± 78	280 ± 20	*0.121*	*0.033*	*0.523*
Phe	130 ± 14	119 ± 12	163 ± 16	147 ± 17	*0.065*	*0.382*	*0.864*
Ile	133 ± 25	152 ± 32	171 ± 36	129 ± 9.7	*0.781*	*0.676*	*0.295*
Leu	344 ± 56	317 ± 39	419 ± 80	297 ± 21	*0.612*	*0.183*	*0.393*
Orn	40.6 ± 12.5	22.9 ± 3.5	39.1 ± 10.9	21.2 ± 1.7	*0.854*	*0.054*	*0.989*
Lys	298 ± 19	245 ± 45	290 ± 45	216 ± 24	*0.608*	*0.095*	*0.770*

Values are means ± SEM, n = 5 per treatment group.

^a-b^Means in a row without a common superscript letter differ (*P* < 0.05) as analyzed by the SNK multiple comparison test.

Cit, citrulline; Orn, ornithine; Tau, taurine.^1^Probability values are indicated in italics.

### 3.3 Net ROS release in leukocytes

#### 3.3.1 Mitogen activation to enhance O_2_^–^ and H_2_O_2_ production

**Figure 2:**
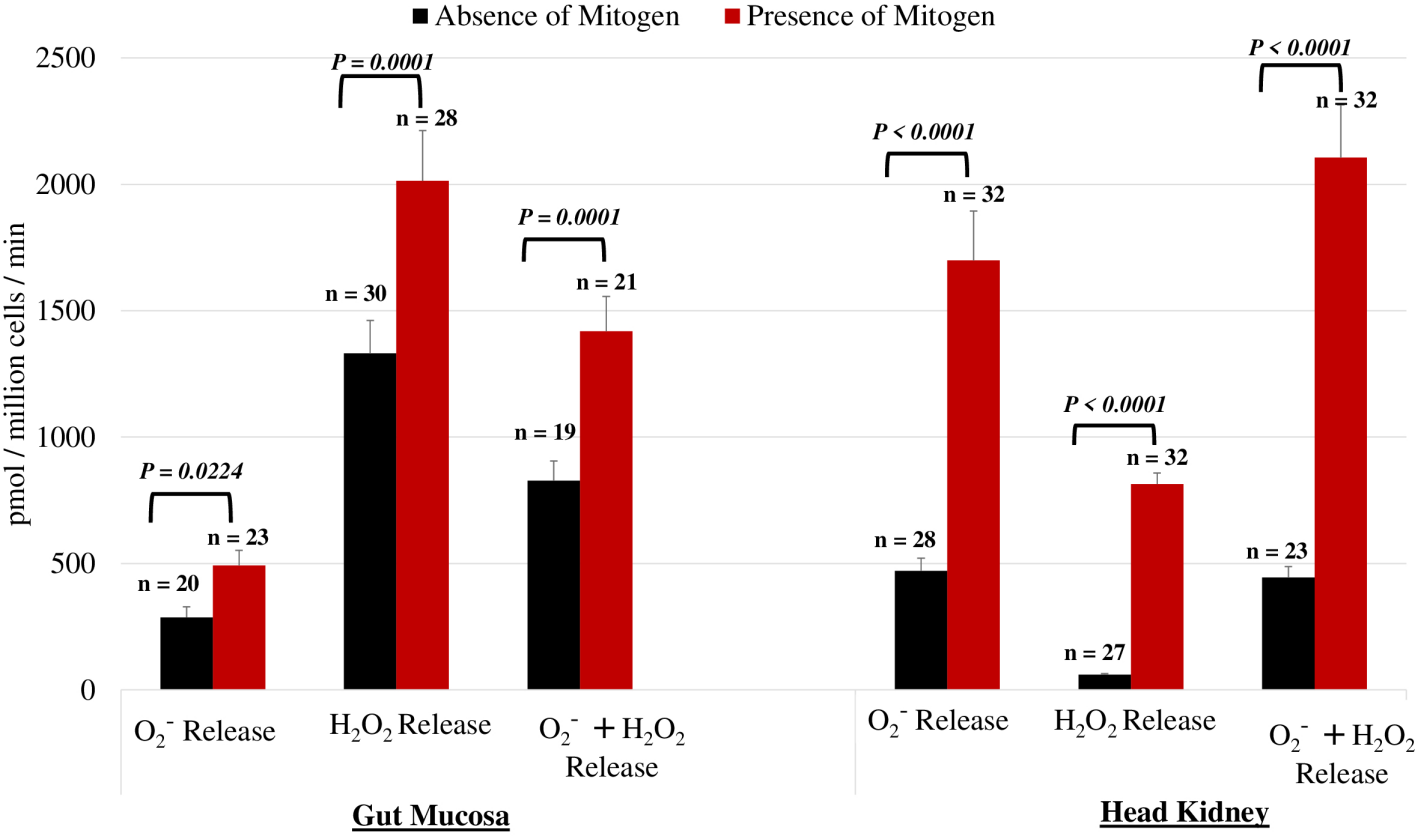
Impact of PMA on O_2_^−^, H_2_O_2_, and their combined levels within the gut mucosa and head kidney leukocytes. Comparison of experimental conditions using the mitogen PMA plus Iono on O_2_^−^ and H_2_O_2_ in leukocytes from the gut mucosa and the head kidney. The results confirmed that the mitogens significantly increased the net release of both O_2_^−^ and H_2_O_2_. leukocytes within both the gut mucosa and the head kidney exhibited a significant increase in activation when stimulated with the mitogen (*P* < 0.05). Differences in values between the presence and absence of mitogens were analyzed by the paired T-test.

To validate the assay, we assessed the effectiveness of PMA + IONO in leukocytes from the gut mucosa and head kidney by measuring ROS production. As illustrated in **Figure 2**, leukocytes from both the gut mucosa and the head kidney exhibited an increase in respiratory burst when stimulated with the mitogens (*P* < 0.05). This robust response underscores the effectiveness and reliability of our assay in triggering and detecting cellular activation. The consistent and pronounced stimulation observed across both tissue types confirms the assay’s sensitivity and specificity in eliciting an immune response, validating its use in further investigations of leukocyte function and activation pathways.

#### 3.3.2 Net release of O_2_^−^ and H_2_O_2_

The data on the net release of O_2_^−^ by HSB leukocytes incubated without mitogen stimulation are summarized in **Table 5**. In intestinal mucosal leukocytes incubated without mitogens, Glu supplementation and LPS challenge increased (*P* < 0.01) the net release of H_2_O_2_ by 40% and 101%, respectively, but had no effect on the net release of O_2_^−^; LPS challenge increased (*P* < 0.05) the net release of O_2_^−^ plus H_2_O_2_ by 52%. There was no interaction effect (*P* > 0.05) in the net release of ROS between Glu supplementation and LPS challenge. In head-kidney leukocytes incubated without mitogens, Glu supplementation decreased the net release of O_2_^−^ and O_2_^−^ plus H_2_O_2_ by 30% (*P* < 0.001) and 21% (*P* < 0.05), respectively, whereas LPS challenge increased (*P* < 0.01) the net release of O_2_^−^, H_2_O_2_, and O_2_^−^ plus H_2_O_2_ by 112% (*P* < 0.001), 37% (*P* < 0.05), and 94% (*P* < 0.001), respectively. There were interaction effects in the net release of O_2_^−^ (*P* < 0.001) and O_2_^−^ plus H_2_O_2_ (*P* < 0.001) between Glu supplementation and LPS challenge.

**Table 5:** Effects of diet and LPS on the net of O_2_^−^ and H_2_O_2_ by the intestinal mucosa and head kidney leukocytes from HSB in the absence of mitogens.

Release	Control		5% Glu		^1^ *P-value*		
	Sham	LPS	Sham	LPS	Glu	LPS	Glu × LPS
Intestinal Mucosal Leukocytes (pmol/10^6^ cells/min)							
O_2_^−^	(n = 5)	(n = 5)	(n = 5)	(n = 3)			
	341 ± 52	355 ± 137	169 ± 27	376 ± 101	*0.278*	*0.305*	*0.312*
H_2_O_2_	(n = 8)	(n = 7)	(n = 7)	(n = 8)			
	522 ± 41^b^	1710 ± 120^a^	1260 ± 107^a^	1870 ± 298^a^	*0.008*	*<0.001*	*0.113*
^*^O_2_^−^ + H_2_O_2_	(n = 5)	(n = 4)	(n = 4)	(n = 3)			
	631 ± 36	1120 ± 177	838 ± 56	1110 ± 255	*0.425*	*0.012*	*0.449*
Head Kidney Leukocytes (pmol/10^6^ cells/min)							
O_2_^−^	(n = 8)	(n = 8)	(n = 8)	(n = 4)			
	251 ± 17^c^	840 ± 73^a^	344 ± 21^b,c^	424 ± 28^b^	*0.001*	*<0.001*	*<0.001*
H_2_O_2_	(n = 6)	(n = 5)	(n = 8)	(n = 8)			
	58.6 ± 13.3	72.3 ± 8.8	44.6 ± 5.8	69.1 ± 2.6	*0.316*	*0.016*	*0.501*
^*^O_2_^−^ + H_2_O_2_	(n = 6)	(n = 5)	(n = 8)	(n = 4)			
	266 ± 16^c^	774 ± 85^a^	366 ± 19^b,c^	455 ± 28^b^	*0.023*	*<0.001*	*<0.001*

Values are means ± SEM with the number of independent observations indicated in the parentheses.

^a-c^Means in a row without a common superscript letter differ (*P* < 0.05) as analyzed by the SNK multiple comparison test.

^*^The sum of O_2_^−^ and ½ H_2_O_2_, based on the principle that every two molecules of O_2_^−^ result in one molecule of H_2_O_2_ (2 O_2_^−^ + 2H^+^ → H_2_O_2_ + O_2_ via superoxide dismutase).^1^Probability values are indicated in italics.

The data on the net release of O_2_^−^ by HSB leukocytes incubated with mitogen stimulation are summarized in **Table 6**. Most of results regarding the effects of Glu supplementation and LPS challenge on ROS release by these cells were qualitatively similar to those for the absence of mitogens. Specifically, in intestinal mucosal leukocytes incubated with mitogens, Glu supplementation and LPS challenge increased (P < 0.01) the net release of H_2_O_2_ by 45% and 83%, respectively, but had no effect on the net release of O_2_^−^. LPS, but not Glu supplementation, increased (P < 0.05) the net release of O_2_^−^ plus H_2_O_2_ by intestinal mucosal leukocytes. There was no interaction effect (P > 0.05) in the net release of ROS by these cells between Glu supplementation and LPS challenge. In head-kidney leukocytes incubated with mitogens, Glu supplementation increased the net release of O_2_^−^, H_2_O_2_, and O_2_^−^ plus H_2_O_2_ by 312% (P < 0.001), 29% (P < 0.05), and 213% (P < 0.001), respectively, whereas LPS challenge increased (P < 0.01) the net release of O_2_^−^, H_2_O_2_, and O_2_^−^ plus H_2_O_2_ by 25% (P < 0.001), 22% (P < 0.05), and 25% (P < 0.001), respectively. There were interaction effects in the net release of O_2_^−^ (P < 0.001) and O_2_^−^ plus H_2_O_2_ by head-kidney leukocytes (P < 0.05) between Glu supplementation and LPS challenge.

**Table 6:** Effects of diet and LPS on the net of O_2_^−^ and H_2_O_2_ by the leukocytes of the intestinal mucosa and head kidney from HSB in the prescence of mitogens.

Release	Control	5% Glu	^1^ *P-value*				
	Sham	LPS	Sham	LPS	Glu	LPS	Glu × LPS
Intestinal Mucosal Leukocytes (pmol/10^6^ cells/min)									
O_2_^−^	(n = 6)	(n = 7)	(n = 4)	(n = 6)			
	522 ± 142	508 ± 146	470 ± 115	456 ± 72	*0.689*	*0.912*	*1.000*
H_2_O_2_	(n = 8)	(n = 7)	(n = 8)	(n = 8)			
	1190 ± 78^b^	1930 ± 72^b^	1510 ± 177^b^	3010 ± 526^a^	*0.020*	*0.001*	*0.210*
^*^O_2_^−^ + H_2_O_2_	(n = 6)	(n = 6)	(n = 4)	(n = 6)			
	1110 ± 164	1370 ± 161	1320 ± 73	1850 ± 389	*0.128*	*0.145*	*0.593*
Head Kidney Leukocytes (pmol/10^6^ cells/min)									
O_2_^−^	(n = 8)	(n = 8)	(n = 8)	(n = 8)			
	317 ± 14^c^	1010 ± 112^b^	2700 ± 63^a^	2770 ± 100^a^	*<0.001*	*<0.001*	*0.001*
H_2_O_2_	(n = 8)	(n = 8)	(n = 8)	(n = 8)			
	688 ± 68^b^	737 ± 109^b^	783 ± 77^b^	1050 ± 16^a^	*0.012*	*0.046*	*0.164*
^*^O_2_^−^ + H_2_O_2_	(n = 8)	(n = 8)	(n = 8)	(n = 8)			
	661 ± 43^c^	1380 ± 166^b^	3090 ± 101^a^	3290 ± 102^a^	*<0.001*	*<0.001*	*0.029*

Values are means ± SEM with the number of independent observations indicated in the parentheses.

^a-c^Means in a row without a common superscript letter differ (*P* < 0.05) as analyzed by the SNK multiple comparison test.

^*^The sum of O_2_^−^ and ½ H_2_O_2_, based on the principle that every two molecules of O_2_^−^ result in one molecule of H_2_O_2_ (2 O_2_^−^ + 2H^+^ → H_2_O_2_ + O_2_ via superoxide dismutase).^1^Probability values are indicated in italics.

### 3.4 Gene expression

LPS challenge did not affect gene expression for IL-1β, TNF-α, and IgT across the head kidney, spleen, and gut mucosa (**Table 7**). In contrast, Glu supplementation upregulated the expression of IL-1β and TNF-α (inflammatory cytokines) in the intestinal mucosa by 374% (*P* < 0.01) and 181% (*P* < 0.05), respectively. Additionally, the expression of IgT in the intestinal mucosa was increased (*P* < 0.05) by 96% increase in Glu-supplemented fish as compared with fish without Glu supplementation.

**Table 7:** Effects of diet and LPS on the relative mRNA expression for the following gene of interest (GOI) IL-1β, TNF-α, and IgT in the head kidney, spleen, and gut mucosa.

GOI	Tissue	Control		5% Glutamate		^1^ *P-value*		
		Sham	LPS	Sham	LPS	Glu	LPS	Glu × LPS
IL-1β	Head Kidney	(n = 6)	(n = 6)	(n = 6)	(n = 6)			
		1.00 ± 0.27	1.15 ± 0.34	1.42 ± 0.36	1.51 ± 0.23	*0.217*	*0.696*	*0.912*
	Spleen	(n = 6)	(n = 5)	(n = 5)	(n = 6)			
		1.00 ± 0.15	1.07 ± 0.35	0.59 ± 0.23	0.62 ± 0.11	*0.062*	*0.806*	*0.925*
	Gut Mucosa	(n = 5)	(n = 5)	(n = 5)	(n = 5)			
		1.00 ± 0.30^b^	0.68 ± 0.05^b^	3.93 ± 1.46^a^	4.03 ± 0.91^a^	*0.002*	*0.897*	*0.813*
TNF-α	Head Kidney	(n = 6)	(n = 6)	(n = 6)	(n = 6)			
		1.00 ± 0.27	1.04 ± 0.45	1.28 ± 0.29	1.26 ± 0.18	*0.427*	*0.980*	*0.927*
	Spleen	(n = 6)	(n = 5)	(n = 6)	(n = 6)			
		1.00 ± 0.21	0.85 ± 0.32	0.43 ± 0.14	0.62 ± 0.12	*0.059*	*0.888*	*0.412*
	Gut Mucosa	(n = 5)	(n = 5)	(n = 5)	(n = 5)			
		1.00 ± 0.39	0.60 ± 0.08	2.31 ± 1.00	2.19 ± 0.44	*0.024*	*0.655*	*0.813*
IgT	Head Kidney	(n = 6)	(n = 6)	(n = 6)	(n = 6)			
		1.00 ± 0.38	1.28 ± 0.46	1.27 ± 0.29	1.18 ± 0.31	*0.808*	*0.783*	*0.585*
	Spleen	(n = 6)	(n = 5)	(n = 6)	(n = 6)			
		1.00 ± 0.29	1.37 ± 0.55	0.91 ± 0.27	1.30 ± 0.20	*0.847*	*0.251*	*0.983*
	Gut Mucosa	(n = 5)	(n = 5)	(n = 5)	(n = 5)			
		1.00 ± 0.29^a^	0.37 ± 0.05^b^	1.32 ± 0.31^a^	1.36 ± 0.19^a^	*0.013*	*0.228*	*0.166*

Values are means ± SEM with the number of independent observations indicated in the parentheses, normalized to the control-sham group.

^a-b^Means in a row without a common superscript letter differ (*P* < 0.05) as analyzed by the SNK multiple comparison test.^1^Probability values are indicated in italics.

### 3.5 Histology

**Figure 3:**
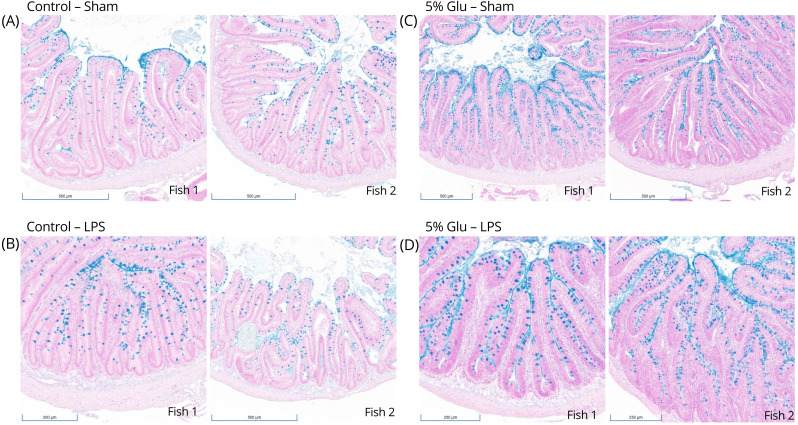
**(A–D)** Goblet cell distribution along villi of the proximal intestine of HSB. Alcian Blue staining demonstrated qualitative differences in goblet cell distribution along the intestinal villi following Glu supplementation. Goblet cells were less apparent in the control-sham HSB than in the 5%-Glu-sham HSB. LPS stimulation also seems to increase the distribution compared to their sham counterparts, indicating a possible mucosal response to the inflammatory stimulus.

Morphological changes in the intestine were observed between the control and Glu-supplemented HSB. Alcian Blue staining revealed qualitative differences in the distribution and abundance of goblet cells along the intestinal villi following Glu supplementation (**Figure 3**). Goblet cells appeared to be less abundant in the control-sham HSB than in the 5%-Glu-sham HSB. LPS stimulation also appeared to increase the abundance of these cells as compared to their sham counterparts, indicating a possible mucosal response to Glu supplementation and the inflammatory stimulus.

## 4 Discussion

Proper redox balance supports mucosal immunity by controlling ROS production, reducing inflammation, and promoting gut health (15). This study examined the effects of dietary Glu supplementation on ROS production and immune function in HSB. To the best of available knowledge, no previous research has directly addressed the impact of dietary Glu in HSB. By exploring Glu’s role in modulating immune responses and oxidative stress, especially in the intestinal mucosa, we set the stage for future research to improve disease resistance and aquaculture sustainability. Several findings warrant discussion.

This is the first study to report plasma AA concentrations for HSB fed a purified AA diet. Dietary Glu supplementation increased the plasma concentrations of Glu, aspartate, asparagine, citrulline, arginine, and tyrosine in HSB without LPS challenge but had no effect on those AAs in LPS-challenged fish (**Table 4**). The gut, liver, skeletal muscle, and kidneys of HSB extensively degrade Glu to form aspartate and asparagine in the absence of LPS (3, 41), but LPS may interfere with these pathways. In omnivorous mammals such as pigs, humans and rats, the enterocytes of the small intestine convert Glu into citrulline and arginine (9), but it is unknown whether arginine-synthetic pathways occur in HSB. Alternatively, an increase in dietary Glu provision reduces the catabolism of arginine by inhibiting the activity of ornithine aminotransferase (i.e., ornithine + α-ketoglutarate → pyrroline-5-carboxylate + Glu) via the mechanism of feedback inhibition (14). This would be of importance in immune responses because Arg is involved in the production of nitric oxide (NO, a nitrogen reactive species) to combat pathogens (42). Likewise, Glu decreased the degradation of tyrosine by inhibiting tyrosine transaminase (tyrosine + α-ketoglutarate → 4-hydroxyphenylpyruvate + Glu) (14). As a direct precursor of catecholamine (epinephrine and nor epinephrine), thyroid hormones (triiodothyronine and thyroxine), dopamine, and melanin, tyrosine can regulate the proliferation of leukocytes and humoral immune responses in mammals (43) and possibly in aquatic animals. In support of this view, tyrosine supplementation has been reported to improve the health and survival of seabream larvae (Diplodus sargus) (44), as well as Gilthead seabreams (Sparus aurata) and meagres (Argyrosomus regius) (45). Further studies are needed to explore Glu’s effects on these specific AAs as well as NO and hormone production. Taurine is highly abundant in red blood cells and tissues of animals, including terrestrial mammals and birds, as well as fish (14). As recently reported by our group (46, 47), the concentration of taurine is unusually high (1 to 2 mM) in the plasma of HSB (**Table 4**). The decrease in the circulating level of taurine with Glu supplementation in the absence of LPS challenge (**Table 4**) suggested an improvement in the integrity of cells (e.g., red blood cells) and tissues (e.g., intestine and liver) possibly due to an increased synthesis of glutathione (a potent antioxidant in cells), thereby reducing the leaking of intracellular taurine into the plasma. Intriguingly, the effect of LPS in reducing plasma taurine concentration possibly by activating taurine removal and excretion via immune responses (e.g., the formation of N-chlorotaurine and N-bromotaurine from taurine plus hypochlorous acid or hypobromous acid) (14) was prevented by Glu supplementation. As taurine plays an important role in antioxidative and anti-inflammatory responses (14), this AA may mediate, in part, the beneficial effects of Glu in HSB. Further studies are warranted to test these novel hypotheses.

Another novel observation from this study is that dietary Glu supplementation promoted weight gain in non-stressed HSB (**Table 3**), whereas immune stimulant reduced growth, consistent with previous studies showing LPS challenges hindered growth in rats (48) and rainbow trout (49). HSB fed a control diet and exposed to the LPS showed lower weight gain than their sham counterparts, confirming that bacterial endotoxins negatively impact fish growth. Notably, HSB supplemented with 5% Glu and not immune-challenged showed the highest weight gain, suggesting Glu supported protein deposition in non-stressed conditions. Studies in pigs (50, 51), and fish (18, 19) have demonstrated that dietary Glu enhances whole-body growth without immune stress, which should be further explored in HSB. Studies in zebrafish have also shown how the route of delivery of the immune stimulant delivery route can affect response (52), suggesting that both the timing and delivery should be experimentally examined.

This study revealed for the first time that Glu increased net ROS release head kidney leukocytes regardless of LPS and in gut mucosal leukocytes in response to LPS challenge. The presence of ROS in the cells could trigger the biochemical reactions for respiratory burst, particularly in leukocytes from LPS-injected HSB. This suggests Glu supports oxidant responses in the gut to kill pathogens and may also have additional systemic effects, as seen in enhanced ROS production in the head kidney during immune activation. These tissue-specific responses highlight Glu’s dual role in regulating oxidative stress and immune function. Similar findings in Jian carp support the view that dietary Glu can enhance ROS scavenging and reduce oxidative damage (19). In this context, superoxide dismutase in the endogenous scavenger catalyzes the dismutation of reactive O_2_^−^ to a more stable non-radical ROS, H_2_O_2_ (53, 54). O_2_^−^ may be more selective in respiratory bursts, while H_2_O_2_ is the main molecule for immune defense. H_2_O_2_ is produced by leukocytes as part of their respiratory burst response involved through phagocytosis (55–57). Phagocytosis is one of fish’s most important defense mechanisms to protect themselves from aquatic microbes. ROS are short-lived, meaning free radicals get rapidly converted into a more favorable ROS, like H_2_O_2,_ to combat bacterial pathogens by adjusting the phagolysosome pH during respiratory burst (53, 54, 58, 59). This would be consistent with the findings on the net release of ROS by neutrophils and monocytes of zebrafish (60), these cells could be the primary immune cells in intestinal mucosal and head-kidney leukocytes of HSB to release H_2_O_2_ and superoxide anion. One key pathway regulating the oxidant-antioxidant balance is the activation of NADPH oxidase, which catalyzes the conversion of molecular oxygen (O_2_) to O_2_− using NADPH as the electron donor (61–64). Glu could facilitate NADPH production and provide energy via ATP formation to the intestinal mucosal and head-kidney leukocytes by promoting the pentose cycle and mitochondrial electron transport.

Additionally, IL-1β expression was increased in the gut mucosa of HSB fed the 5% Glu diet, suggesting tissue-specific immune modulation. Extensive Glu catabolism occurs in the intestinal mucosa of humans (65), rats (61, 62) and piglets (7, 63). High concentrations of H_2_O_2_ were observed in HSB’s gut mucosal leukocytes due to Glu supplementation, which could activate downstream inflammatory responses. High concentrations of H_2_O_2_ could also stimulate thioredoxin-interacting proteins, thereby facilitating pore formation in the plasma membrane and releasing IL-1β as seen in mice (66) and zebrafish (67). Furthermore, the controlled interactions of H_2_O_2_ with target pathogens could be due to its diffusing properties, making it more typical for cell growth and maturation within the gut mucosa (59, 66–70). This is probably why there was an increase in the abundance of goblet cells along the intestinal epithelium of Glu-supplemented HSB (**Figure 3**). The formation and migration of these cells from the crypt to the villus could be powered partly by Glu-derived energy.

Simultaneously, both TNF-α and IL-1β expression increased in the intestinal mucosa of Glu-supplemented HSB (**Table 7**). While these pro-inflammatory cytokines are typically co-expressed, TNF-α expression can be modulated by cGMP that is formed from GTP by guanylyl cyclase in response to stimuli such as the free radical NO (68, 69). NO has the ability to react with both O_2_^−^ and H_2_O_2_ to form ONOO^−^, which can amplify the oxidative mechanism to kill pathogens and can also downregulate inflammation by inhibiting the NF-κB pathway and, therefore, reduce the expression of TNF-α (56, 71–74). Most cell types can produce TNF-α, particularly M1-activated macrophages (71, 75, 76), as confirmed in an in-vivo study in transgenic zebrafish (77). However, in most teleost fish species, there are multiple isoforms of TNF-α, indicating functional specialization that allows a more nuanced control of macrophage activation and inflammation resolution (78, 79). Isoforms of TNF-α may differentially regulate the extent of M1 polarization and NO production, and their distinct roles could balance pro-inflammatory and anti-inflammatory responses, particularly in teleost fish with multiple TNF-α variants. Thus, further research on isoform-specific functions, particularly in fish models, could provide valuable evolutionary insights and reveal mechanisms critical to innate immunity and inflammation resolution while illuminating broader principles of immune regulation and inflammation across species.

IgT expression was elevated with Glu supplementation, compared to the control group, supporting our hypothesis. This is an exciting finding because this is the first study to investigate the effect of dietary Glu on IgT at the mRNA level. As mentioned previously, Glu could enhance NADPH production and also provide ATP to the intestinal mucosa leukocytes by both allosterically activating key enzymes of the pentose cycle and promoting electron transport through the rapid conversion of O_2_− to H_2_O_2_ within the gut mucosa. Therefore, AAs must have sufficient bioavailability for B lymphocytes to proliferate, such as Glu (15, 28). IgT may be the key molecule to prevent mucosal damage caused by ROS, but further studies are needed to properly quantify IgT production at the protein level. These studies should contribute to the emerging understanding of IgT’s roles in distinct teleost immune tissues (80) in the bigger picture of fish primary and secondary lymphoid tissues (81). As mentioned, the immune stimulant’s delivery route and the timing of supplementation can affect response. As seen in Carvalho et al. (82), supplementing with dietary methionine for four weeks and a bacterial bath challenge decreased proinflammatory mRNA expression within the head kidney. It would be worth investigating the effects of shorter supplementation periods and even challenges with bacteria that pose a threat to aquaculture farms.

Lastly, the increased abundance and distribution of goblet cells in the intestine (**Figure 3**) could indicate that Glu-supplementation can modulate mucosal immunity, for they are typical morphological markers to identify an active mucosal immune response (15). Glu has been shown to increase gut integrity (83) and enhance mucosal barrier function (7), which could be done through the production of mucin (84). Further exploration is needed to determine if dietary Glu directly or indirectly impacts mucin production. There was increased distribution of goblet cells in response to LPS stimulation (**Figure 3**), which has been shown to upregulate mucin and cytokine mRNA expression in teleost fish (85). Mucin production can be associated with inflammation caused by a bacterial infection, but it is a needed defense mechanism (85, 86). This makes the possibility of supplementation with Glu appealing, for it provides a non-invasive method to modulate the immune response. Thus, dietary Glu is essential for the intestinal mucosal immune response in juvenile HSB, and this AA may also serve as an effective adjuvant for vaccination in fish. Based on our findings on the beneficial effects of dietary Glu on HSB growth and net ROS release, particularly hydrogen peroxide (H_2_O_2_) in the intestinal mucosa, further investigation of the relationship between Glu and redox balance in intestinal mucosal immunity should offer valuable insights for optimizing health to advance global aquaculture and promote the sustainable production of high-quality food for human consumption.

**Figure 4:**
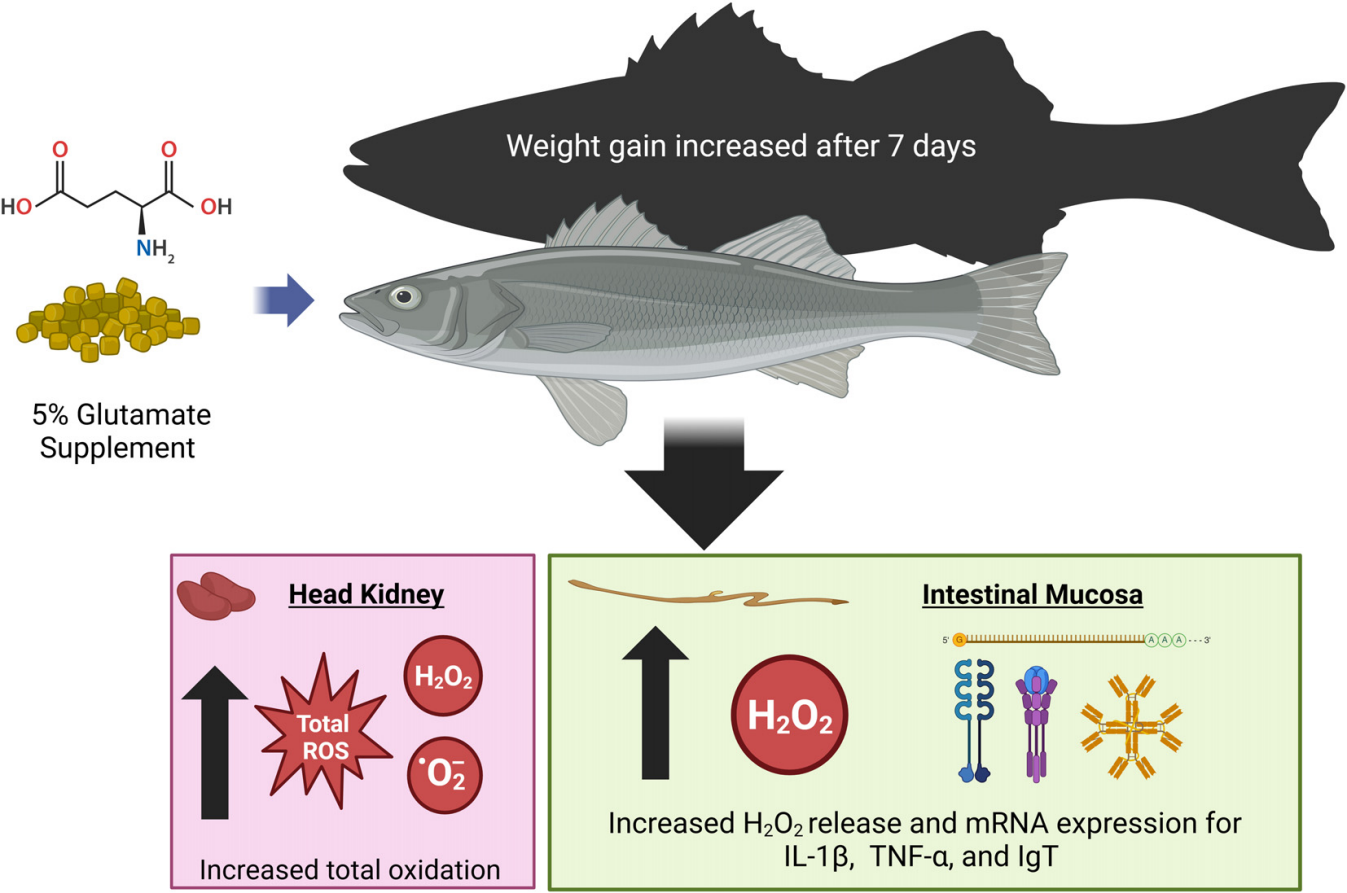
A summary for the effect of dietary glutamate supplementation on improving growth and leukocyte respiratory burst in hybrid striped bass. Dietary supplementation with 5% Glu may modulate intestinal mucosal immunity by increasing the production of O_2_^−^ and H_2_O_2_ and improve whole-body growth by increasing intracellular protein accretion in HSB to enhance disease resistance. Dietary Glu is essential for the intestinal mucosal immune response in juvenile HSB, and this functional amino acid may also serve as an effective adjuvant for vaccination in fish. Created in BioRender. Hissen, K. (2025) https://BioRender.com/p33v886.

Overall, this study underscores the importance of redox mechanisms in immune function, particularly regarding AA nutrition and gut health in aquatic species (**Figure 4**). The findings have significant implications for aquaculture, highlighting the potential benefits of high-Glu diets in promoting fish growth and immune responses. Further research is needed to clarify the metabolic and physiological pathways through which Glu enhances fish growth. Additionally, these results suggest that using a purified diet is a reliable and useful approach for immunonutrition studies in HSB, allowing targeted nutrient manipulation for evaluating immune regulation and overall performance.

## 5 Conclusions

This study highlights the role of dietary Glu supplementation in modulating gut mucosal leukocytes in teleost fish, such as HSB. Dietary Glu could influence key mechanisms, including H_2_O_2_ generation, and regulate cellular signaling for redox balance. Findings from this study show that Glu supplementation enhances growth, modulates oxidative stress, and supports immune function, particularly by reducing oxidative stress in the gut while increasing immune activity in the intestinal mucosa and head kidney. These results suggest that Glu can be an effective immunonutrient in both stressed and non-stressed environments. However, further research is needed to clarify the pathways through which Glu influences immune function and growth. Optimizing its use in high-density aquaculture settings could improve fish health and productivity, positively impacting sustainable food production in response to growing global demand.

## Data Availability

The original contributions presented in the study are included in the article. Further inquiries can be directed to the corresponding authors.

## References

[1] Wu G. Dietary protein intake and human health. Food Funct. (2016) 7:1251–65. doi: 10.1039/c5fo01530h 26797090

[2] Tomé D. Digestibility issues of vegetable versus animal proteins: protein and amino acid requirements—Functional aspects. Food Nutr Bull. (2013) 34:272–4. doi: 10.1177/156482651303400225 23964409

[3] Li X Zheng S Wu G. Nutrition and metabolism of glutamate and glutamine in fish. Amino Acids. (2020) 52:671–91. doi: 10.1007/s00726-020-02851-2 32405703

[4] Tomé D. The roles of dietary glutamate in the intestine. Ann Nutr Metab. (2018) 73:15–20. doi: 10.1159/000494777 30508814

[5] Burrin DG Stoll B. Metabolic fate and function of dietary glutamate in the gut. Am J Clin Nutr. (2009) 90:850S–6S. doi: 10.3945/ajcn.2009.27462Y 19587091

[6] Hou Y Yin Y Wu G. Dietary essentiality of “nutritionally non-essential amino acids” for animals and humans. Exp Biol Med. (2015) 240:997–1007. doi: 10.1177/1535370215587913 PMC4935284 26041391

[7] Jiao N Wu Z Ji Y Wang B Dai Z Wu G. L-Glutamate enhances barrier and antioxidative functions in intestinal porcine epithelial cells1,2. J Nutr. (2015) 145:2258–64. doi: 10.3945/jn.115.217661 26338884

[8] Hou Y Wu G. L-Glutamate nutrition and metabolism in swine. Amino Acids. (2018) 50:1497–510. doi: 10.1007/s00726-018-2634-3 30116978

[9] Wu G. Intestinal mucosal amino acid. J Nutr. (1998) 128:1249–52. doi: 10.1093/jn/128.8.1249 9687539

[10] Van Waarde A. Aerobic and anaerobic ammonia production by fish. Comp Biochem Physiol B. (1983) 74:675–84. doi: 10.1016/0305-0491(83)90127-x 6345084

[11] Wu G Marliss EB. Enhanced glucose metabolism and respiratory burst in peritoneal macrophages from spontaneously diabetic BB rats. Diabetes. (1993) 42:520–9. doi: 10.2337/diab.42.4.520 8384132

[12] Wu G. Principles of Animal Nutrition. Boca Raton, FL: CRC Press (2017). p. 1–772. doi: 10.1201/9781315120065

[13] Leto TL Geiszt M. Role of nox family NADPH oxidases in host defense. Antioxid Redox Signal. (2006) 8:1549–61. doi: 10.1089/ars.2006.8.1549 16987010

[14] Wu G. Amino Acids: Biochemistry and Nutrition. 2nd ed. Boca Raton, FL: CRC Press (2022) p. 1–788. doi: 10.1201/9781003092742

[15] Hissen KL He W Wu G Criscitiello MF. Immunonutrition : facilitating mucosal immune response in teleost intestine with amino acids through oxidant- antioxidant balance. Front Immunol. (2023) 14:1–18. doi: 10.3389/fimmu.2023.1241615 PMC10570457 37841275

[16] He W Hissen KL Crsicitiello MF Wu G. (2023). Pentose cycle activity in intestinal mucosal leukocytes of hybrid striped bass, in: 2023 Conference of Research Workers in Animal Diseases, Chicago, IL. 14, p. 401.

[17] Wu G. Functional amino acids in growth, reproduction, and health. Adv Nutr. (2010) 1:31–7. doi: 10.3945/an.110.1008 PMC3042786 22043449

[18] Wang M Li E Huang Y Liu W Wang S Li W. Dietary supplementation with glutamate enhanced antioxidant capacity, ammonia detoxification and ion regulation ability in Nile tilapia (Oreochromis niloticus) exposed to acute alkalinity stress. Aquaculture. (2025) 594:741360. doi: 10.1016/j.aquaculture.2024.741360

[19] Zhao Y Li JY Yin L Feng L Liu Y Jiang WD. Effects of dietary glutamate supplementation on flesh quality, antioxidant defense and gene expression related to lipid metabolism and myogenic regulation in Jian carp (Cyprinus carpio var. Jian). Aquaculture. (2019) 502:212–22. doi: 10.1016/j.aquaculture.2018.12.050

[20] de Macêdo ÉS Graciano Franco TS Marçal Natali MR Paulovski Panaczevicz PA Rudnik AR Galiotto Miranda JA. Dietary glutamine-glutamate supplementation enhances growth performance and intestinal villi development in cage-farmed Nile tilapia fingerlings. Rev Bras Zootec. (2021) 50:1–10. doi: 10.37496/RBZ5020200010

[21] Hissen KL He W Wu G Criscitiello MF. (2025). Glutamate effects on reactive oxygen species in intestinal mucosal leukocytes of juvenile hybrid striped bass, in: 2025 Conference of Research Workers in Animal Diseases, Chicago, IL. p. 39.

[22] Salinas I. The mucosal immune system of teleost fish. Biol (Basel). (2015) 4:525–39. doi: 10.3390/biology4030525 PMC4588148 26274978

[23] Martínez-López A Tyrkalska SD Alcaraz-Pérez F Cabas I Candel S Martínez Morcillo FJ. Evolution of LPS recognition and signaling: The bony fish perspective. Dev Comp Immunol. (2023) 145:0–3. doi: 10.1016/j.dci.2023.104710 37080369

[24] Lieschke GJ Trede NS. Fish immunology. Curr Biol. (2009) 19:R678–82. doi: 10.1016/j.cub.2009.06.068 19706273

[25] FAO. *The State of World Fisheries and Aquaculture (SOFIA*). Rome: FAO (2022). p. 266.

[26] Suehs BA Iii DMG Wu G. Glycine nutrition and biochemistry from an aquaculture perspective. Animal Frontiers. (2024) 14:17–23. doi: 10.1093/af/vfae014 PMC11377068 39246842

[27] Cheng Z Gatlin DM Buentello A. Dietary supplementation of arginine and/or glutamine influences growth performance, immune responses and intestinal morphology of hybrid striped bass (Morone chrysops×Morone saxatilis). Aquaculture. (2012) 362–363:39–43. doi: 10.1016/j.aquaculture.2012.07.015

[28] Li P He W Wu G. Composition of Amino Acids in Foodstuffs for Humans and Animals. Adv Exp Med Biol. (2021) 1332:189–210. doi: 10.1007/978-3-030-74180-8_11 34251645

[29] Hou Y He W Hu S Wu G. Composition of polyamines and amino acids in plant-source foods for human consumption. Amino Acids. (2019) 51:1153–65. doi: 10.1007/s00726-019-02751-0 31197570

[30] Jobling M. National Research Council (NRC): Nutrient requirements of fish and shrimp. Aquac Int. (2011) 20:601–2. doi: 10.1007/s10499-011-9480-6

[31] Johnston RBJ. Measurement of O2- secreted by monocytes and macrophages. Methods Enzymol. (1984) 105:365–9. doi: 10.1016/s0076-6879(84)05049-7 6328188

[32] Dai Z Wu Z Jia S Wu G. Analysis of amino acid composition in proteins of animal tissues and foods as pre-column o-phthaldialdehyde derivatives by HPLC with fluorescence detection. J Chromatogr B Anal Technol BioMed Life Sci. (2014) 964:116–27. doi: 10.1016/j.jchromb.2014.03.025 24731621

[33] Jeon SJ Fast MD. Age-0 striped bass, Morone saxatilis (Walbaum, 1792), response to immunostimulation. J Appl Ichthyol. (2015) 31:125–34. doi: 10.1111/jai.12665

[34] Byadgi O Beraldo P Volpatti D Massimo M Bulfon C Galeotti M. Expression of infection-related immune response in European sea bass (Dicentrarchus labrax) during a natural outbreak from a unique dinoflagellate Amyloodinium ocellatum. Fish Shellfish Immunol. (2019) 84:62–72. doi: 10.1016/j.fsi.2018.09.069 30266602

[35] Fu WJ Hu J Spencer T Carroll R Wu G. Statistical models in assessing fold change of gene expression in real-time RT-PCR experiments. Comput Biol Chem. (2006) 30:21–6. doi: 10.1016/j.compbiolchem.2005.10.005 16321570

[36] Carnoy JB. Les globules polaires de l’Ascaris clavata. Cellule. (1887) 3:247–324.

[37] Puchtler H Sweat Waldrop F Conner HM Terry MS. Carnoy fixation: practical and theoretical considerations. Histochemie. (1968) 16:361–71. doi: 10.1007/BF00306359 4179106

[38] Blick AK Giaretta PR Sprayberry S Bush-Vadala C Paulk CB Boeckman J. Comparison of 2 fixatives in the porcine colon for in *situ* microbiota studies. J Anim Sci. (2019) 97:4803–9. doi: 10.1093/jas/skz325 PMC6915214 31845740

[39] Assaad HI Hou Y Zhou L Carroll RJ Wu G. Rapid publication-ready MS-Word tables for two-way ANOVA. Springerplus. (2015) 4:33. doi: 10.1186/s40064-015-0795-z 25635246 PMC4305362

[40] Lee U Garcia TP Carroll RJ Gilbreath KR Wu G. Analysis of repeated measures data in nutrition research. FBL. (2019) 24:1377–89. doi: 10.2741/4785 PMC6556387 31136985

[41] Jia S Li X Zheng S Wu G. Amino acids are major energy substrates for tissues of hybrid striped bass and zebrafish. Amino Acids. (2017) 49:2053–63. doi: 10.1007/s00726-017-2481-7 28852872

[42] Wu G Bazer FW Davis TA Woo S Li P Rhoads JM. Arginine metabolism and nutrition in growth, health and disease. Amino Acids. (2009) 37:153–68. doi: 10.1007/s00726-008-0210-y PMC2677116 19030957

[43] Li P Yin YL Li D Kim WS Wu G. Amino acids and immune function. Br J Nutr. (2007) 98:237–52. doi: 10.1017/S000711450769936X 17403271

[44] Saavedra M Conceição LEC Barr Y Helland S Pousão-Ferreira P Yúfera M. Tyrosine and phenylalanine supplementation on Diplodus sargus larvae: Effect on growth and quality. Aquac Res. (2010) 41:1523–32. doi: 10.1111/j.1365-2109.2009.02446.x

[45] Salamanca N Giráldez I Morales E de la Rosa I Herrera M. Phenylalanine and tyrosine as feed additives for reducing stress and enhancing welfare in gilthead seabream and meagre. Animals. (2021) 11:1–11. doi: 10.3390/ani11010045 PMC7824165 33383663

[46] Li X He W Wu G. Dietary glycine supplementation enhances the growth performance of hybrid striped bass (Morone saxatilis ♀× Morone chrysops ♂) fed soybean meal-based diets. J Anim Sci. (2023) 101:skad345. doi: 10.1093/jas/skad345 37801645 PMC10635675

[47] He W Li X Wu G. Dietary glycine supplementation improves the growth performance of 110- to 240-g (phase II) hybrid striped bass (Morone saxatilis ♀× Morone chrysops ♂) fed soybean meal-based diets. J Anim Sci. (2023) 101:skad400. doi: 10.1093/jas/skad400 38038705 PMC10734566

[48] Iwasa T Matsuzaki T Kinouchi R Fujisawa S Murakami M Kiyokawa M. Neonatal LPS injection alters the body weight regulation systems of rats under non-stress and immune stress conditions. Int J Dev Neurosci. (2010) 28:119–24. doi: 10.1016/j.ijdevneu.2009.08.015 19733650

[49] Shepherd BS Spear AR Philip AM Leaman DW Stepien CA Sepulveda-Villet OJ. Effects of cortisol and lipopolysaccharide on expression of select growth-, stress- and immune-related genes in rainbow trout liver. Fish Shellfish Immunol. (2018) 74:410–8. doi: 10.1016/j.fsi.2018.01.003 29325711

[50] Yin J Liu M Ren W Duan J Yang G Zhao Y. Effects of dietary supplementation with glutamate and aspartate on diquat-induced oxidative stress in pigletse. PloS One. (2015) 10:1–11. doi: 10.1371/journal.pone.0122893 PMC4398417 25875335

[51] Zhao Y Zhang T-R Li Q Feng L Liu Y Jiang W-D. Effect of dietary L-glutamate levels on growth, digestive and absorptive capability, and intestinal physical barrier function in Jian carp (Cyprinus carpio var. Jian). Anim Nutr (Zhongguo xu mu shou yi xue hui). (2020) 6:198–209. doi: 10.1016/j.aninu.2020.02.003 PMC7283372 32542201

[52] Weir H Chen PL Deiss TC Jacobs N Nabity MB Young M. DNP-KLH yields changes in leukocyte populations and immunoglobulin isotype use with different immunization routes in zebrafish. Front Immunol. (2015) 6:606. doi: 10.3389/fimmu.2015.00606 26648935 PMC4664633

[53] Dickinson BC Chang CJ. Chemistry and biology of reactive oxygen species in signaling or stress responses. Nat Chem Biol. (2011) 7:504–11. doi: 10.1038/nchembio.607 PMC3390228 21769097

[54] Jayavelu AK Moloney JN Böhmer F-D Cotter TG. NOX-driven ROS formation in cell transformation of FLT3-ITD-positive AML. Exp Hematol. (2016) 44:1113–22. doi: 10.1016/j.exphem.2016.08.008 27666490

[55] Baier-Anderson C Anderson RS. Suppression of superoxide production by chlorothalonil in striped bass (Morone saxatilus) macrophages: The role of cellular sulfhydryls and oxidative stress. Aquat Toxicol. (2000) 50:85–96. doi: 10.1016/S0166-445X(99)00092-2 10930652

[56] Rieger AM Konowalchuk JD Grayfer L Katzenback BA Havixbeck JJ Kiemele MD. Fish and mammalian phagocytes differentially regulate pro-inflammatory and homeostatic responses *in vivo* . PloS One. (2012) 7:10. doi: 10.1371/journal.pone.0047070 PMC3479104 23110059

[57] Rieger AM Hall BE Barreda DR. Macrophage activation differentially modulates particle binding, phagocytosis and downstream antimicrobial mechanisms. Dev Comp Immunol. (2010) 34:1144–59. doi: 10.1016/j.dci.2010.06.006 20600280

[58] Criscitiello MF Dickman MB Samuel JE de Figueiredo P. Tripping on acid: trans-kingdom perspectives on biological acids in immunity and pathogenesis. PloS Pathog. (2013) 9:7. doi: 10.1371/journal.ppat.1003402 PMC3715416 23874196

[59] Gao F Zhao Y Shi X Qiao D Pei C Kong X. Signalling regulation of reactive oxygen species in fish inflammation. Rev Aquac. (2024) 16:1266–85. doi: 10.1111/raq.12895

[60] Martínez-Navarro FJ Martínez-Morcillo FJ de Oliveira S Candel S Cabas I García-Ayala A. Hydrogen peroxide in neutrophil inflammation: Lesson from the zebrafish. Dev Comp Immunol. (2020) 105:103583. doi: 10.1016/j.dci.2019.103583 31862296

[61] Windmueller HG Spaeth AE. Intestinal metabolism of glutamine and glutamate from the lumen as compared to glutamine from blood. Arch Biochem Biophys. (1975) 171:662–72. doi: 10.1016/0003-9861(75)90078-8 1200644

[62] Windmueller HG Spaeth AE. Respiratory fuels and nitrogen metabolism *in vivo* in small intestine of fed rats. Quantitative importance of glutamine, glutamate, and aspartate. J Biol Chem. (1980) 255:107–12. doi: 10.1016/S0021-9258(19)86270-1 7350142

[63] Reeds PJ Burrin DG Jahoor F Wykes L Henry J Frazer EM. Enteral glutamate is almost completely metabolized in first pass by the gastrointestinal tract of infant pigs. Am J Physiol - Endocrinol Metab. (1996) 270:413–8. doi: 10.1152/ajpendo.1996.270.3.e413 8638686

[64] Reeds PJ Burrin DG Stoll B Jahoor F. Intestinal glutamate metabolism. J Nutr. (2000) 130:978S–82S. doi: 10.1093/jn/130.4.978s 10736365

[65] Battezzati A Brillon DJ Matthews DE. Oxidation of glutamic acid by the splanchnic bed in humans. Am J Physiol - Endocrinol Metab. (1995) 269:269–76. doi: 10.1152/ajpendo.1995.269.2.e269 7653544

[66] Zhou R Tardivel A Thorens B Choi I Tschopp J. Thioredoxin-interacting protein links oxidative stress to inflammasome activation. Nat Immunol. (2010) 11:136–40. doi: 10.1038/ni.1831 20023662

[67] Yin Y Zhou Z Liu W Chang Q Sun G Dai Y. Vascular endothelial cells senescence is associated with NOD-like receptor family pyrin domain-containing 3 (NLRP3) inflammasome activation via reactive oxygen species (ROS)/thioredoxin-interacting protein (TXNIP) pathway. Int J Biochem Cell Biol. (2017) 84:22–34. doi: 10.1016/j.biocel.2017.01.001 28064010

[68] Chance B Sies H Boveris A. Hydroperoxide metabolism in mammalian organs. Physiol Rev. (1979) 59:527–605. doi: 10.1152/physrev.1979.59.3.527 37532

[69] Lyublinskaya O Antunes F. Measuring intracellular concentration of hydrogen peroxide with the use of genetically encoded H2O2 biosensor HyPer. Redox Biol. (2019) 24:101200. doi: 10.1016/j.redox.2019.101200 31030065 PMC6482347

[70] Wang C Yuan Z Li J Liu Y Li R Li S. Acute effects of antimony exposure on adult zebrafish (Danio rerio): From an oxidative stress and intestinal microbiota perspective. Fish Shellfish Immunol. (2022) 123:1–9. doi: 10.1016/j.fsi.2022.02.050 35219828

[71] Wiegertjes GF Wentzel AS Spaink HP Elks PM Fink IR. Polarization of immune responses in fish: The ‘macrophages first’ point of view. Mol Immunol. (2016) 69:146–56. doi: 10.1016/j.molimm.2015.09.026 26471699

[72] Fang YZ Yang S Wu G. Free radicals, antioxidants, and nutrition. Nutrition. (2002) 18:872–9. doi: 10.1016/S0899-9007(02)00916-4 12361782

[73] McCord JM. The evolution of free radicals and oxidative stress. Am J Med. (2000) 108:652–9. doi: 10.1016/S0002-9343(00)00412-5 10856414

[74] Fridovich I. Fundamental aspects of reactive oxygen species, or what’s the matter with oxygen? Ann N Y Acad Sci. (1999) 893:13–8. doi: 10.1111/j.1749-6632.1999.tb07814.x 10672226

[75] Hardbower DM Asim M Luis PB Singh K Barry DP Yang C. Ornithine decarboxylase regulates M1 macrophage activation and mucosal inflammation via histone modifications. Proc Natl Acad Sci USA (2017) 114(5):751–60. doi: 10.1073/pnas.1614958114 PMC5293075 28096401

[76] Mosser DM Edwards JP. Exploring the full spectrum of macrophage activation. Nat Publ Gr. (2008) 8:958–69. doi: 10.1038/nri2448 PMC2724991 19029990

[77] Nguyen-Chi M Laplace-Builhe B Travnickova J Luz-Crawford P Tejedor G Phan QT. Identification of polarized macrophage subsets in zebrafish. Elife. (2015) 4:e07288. doi: 10.7554/eLife.07288 26154973 PMC4521581

[78] Wang T Secombes CJ. The cytokine networks of adaptive immunity in fish. Fish Shellfish Immunol. (2013) 35:1703–18. doi: 10.1016/j.fsi.2013.08.030 24036335

[79] Wiens GD Glenney GW. Origin and evolution of TNF and TNF receptor superfamilies. Dev Comp Immunol. (2011) 35:1324–35. doi: 10.1016/j.dci.2011.03.031 21527275

[80] Mo Z Lin H Lai X Dan P Wu H Luo X. The predominant role of IgM in grouper (Epinephelus coioides) mucosal defense against ectoparasitic protozoan infection. Fish Shellfish Immunol. (2024) 155:110023. doi: 10.1016/j.fsi.2024.110023 39547269

[81] Mitchell CD Criscitiello MF. Comparative study of cartilaginous fish divulges insights into the early evolution of primary, secondary and mucosal lymphoid tissue architecture. Fish Shellfish Immunol. (2020) 107:435–43. doi: 10.1016/j.fsi.2020.11.006 33161090

[82] Carvalho I Peixoto D Ferreira I Robledo D Ramos-Pinto L Silva RM. Exploring the effects of dietary methionine supplementation on European seabass mucosal immune responses against Tenacibaculum maritimum. Front Immunol. (2025) 16:1513516. doi: 10.3389/fimmu.2025.1513516 39911390 PMC11794538

[83] Vermeulen MAR de Jong J Vaessen MJ van Leeuwen PA Houdijk APJ. Glutamate reduces experimental intestinal hyperpermeability and facilitates glutamine support of gut integrity. World J Gastroenterol. (2011) 17:1569–73. doi: 10.3748/wjg.v17.i12.1569 PMC3070128 21472123

[84] Święch E Tuśnio A Taciak M Barszcz M. Modulation of mucin secretion in the gut of young pigs by dietary threonine and non-essential amino acid levels. Animals. (2022) 12:1–14. doi: 10.3390/ani12030270 PMC8833754 35158594

[85] Smirnova MG Guo L Birchall JP Pearson JP. LPS up-regulates mucin and cytokine mRNA expression and stimulates mucin and cytokine secretion in goblet cells. Cell Immunol. (2003) 221:42–9. doi: 10.1016/S0008-8749(03)00059-5 12742381

[86] Wang J Gao J Sheng X Tang X Xing J Chi H. Teleost Muc2 and Muc5ac: Key guardians of mucosal immunity in flounder (Paralichthys olivaceus). Int J Biol Macromol. (2024) 277:134127. doi: 10.1016/j.ijbiomac.2024.134127 39053833

